# Adenovirus-Vectored Capsid Proteins of the Serotype A Foot-and-Mouth Disease Virus Protect Guinea Pigs Against Challenge

**DOI:** 10.3389/fmicb.2020.01449

**Published:** 2020-07-09

**Authors:** Yinli Xie, Huiyun Chang, Zhiyong Li, Yanming Zhang

**Affiliations:** ^1^College of Veterinary Medicine, Northwest A&F University, Yangling, China; ^2^State Key Laboratory of Veterinary Etiological Biology, OIE/National Foot-and-Mouth Disease Reference Laboratory, Key Laboratory of Animal Virology of Ministry of Agriculture, Lanzhou Veterinary Research Institute, Chinese Academy of Agricultural Sciences, Lanzhou, China

**Keywords:** foot-and-mouth disease virus, capsid proteins, recombinant adenovirus, mutated viral 3C protease, cytokines, IgA antibody

## Abstract

Type A foot-and-mouth disease virus (FMDV) has been detected on China’s pig farms since 2015, and all suspected samples have been strain A/GDMM/CHA/2013. To overcome the shortcomings of inactive FMDV vaccines, we expressed the capsid protein precursor P1-2A and mutated viral 3C protease of FMDV strain A/GDMM/CHA/2013 in a replication-deficient human adenovirus type 5 vector in this study. A significant humoral immune response, T-cell-mediated antiviral response, and mucosa-mediated antiviral response were induced by the adenovirus-vectored FMDV vaccines in BALB/c mice. Immunization of guinea pigs with the adenovirus-vectored FMD vaccines induced significant neutralizing antibodies and anti-FMDV immunoglobulin A antibodies. The recombinant adenovirus rAdv-P12A3C^G38SF48S^-GD protected 100% of guinea pigs against challenge when administered intramuscularly. Our study demonstrated the potential utility of rAdv-P12A3C^G38SF48S^-GD as a vaccine against type A FMDV.

## Introduction

Foot-and-mouth disease (FMD) is an acute, febrile, highly contact-dependent contagious disease of cloven-hoofed animals caused by foot-and-mouth disease virus (FMDV), which belongs to the genus *Aphthovirus* in the family Picornaviridae (Jamal and Belsham, 2013; Knight-Jones et al., 2016). A wide range of animal species is susceptible to FMDV infection, including economically important breeds of cattle, pig, and sheep (Arzt et al., 2017; Arzt and Belsham, 2018; Sobhy et al., 2018). Foot-and-mouth disease virus is a single-stranded, non-enveloped RNA virus. Its genomic RNA is approximately 8.5 kb in length and consists of a single open reading frame that encodes a precursor polyprotein. The precursor protein is cleaved into individual mature proteins by virally encoded proteases. The 3C viral protein is a vital protease that plays an extremely important role in the cleavage of the viral structural proteins, which allows the assembly of the FMDV capsid in infected cells. The individual mature proteins VP1, VP3, and VP0 spontaneously form the 5S protomer, five of which assemble into a 12S pentamer. Twelve pentamers then assemble into the 75S viral capsid (Senthilkumaran et al., 2017; Palinski and Bertram, 2019). The viral capsid has been reported to have very similar antigen specificity as the complete FMDV 146S antigen (Rodriguez and Grubman, 2009; Kushnir et al., 2012; Scotti and Rybicki, 2013).

Foot-and-mouth disease virus has a wide host range, a high rate of genetic variation, and great antigenic differences. It has seven serotypes (A, O, C, Asia1, SAT1, SAT2, and SAT3) and more than 100 serosubtypes (Diab et al., 2019). Many new variants also appear every year. No cross-immunity is induced by the seven serotypes. There is also only a partial cross-immunity between the various subtypes of the same serotype (Robinson et al., 2016b). The variability in and polymorphism of FMDV have made the prevention and control of FMD very difficult. The major serotypes prevalent in China are types A and O. In economically underdeveloped countries, immunization with traditional inactivated vaccines is the principal way of controlling and eradicating FMDV. However, traditional inactivated vaccines have many shortcomings, including thermal instability, short duration of immunization, high cost, strict biosafety procedures required during vaccine production, and incomplete viral inactivation (Robinson et al., 2016a). This prompted us to seek a safer and more effective FMDV vaccine. The development of novel molecular vaccine technologies has prompted novel strategies for the construction of FMD molecular vaccines. Recombinant viral vector vaccines are an important component of molecular vaccine research. The use of adenovirus as a vector to construct a viral vector vaccine expressing the empty FMDV capsid has been extensively studied (Mayr et al., 1999, 2001; Moraes et al., 2002; Wu et al., 2003; Schutta et al., 2016). However, the capsid antigen of FMDV strain A/GDMM/CHA/2013 has never been expressed. The type A FMDVs detected in samples from China’s pig farms since 2015 have all been strain A/GDMM/CHA/2013. Therefore, in this study, we expressed the capsid protein precursor P1-2A and viral protease 3C of FMDV strain A/GDMM/CHA/2013 in a replication-deficient human adenovirus type 5 vector. Amino acid mutations G38S and F48S were introduced in the 3C region to reduce its protease activity, based on an earlier report (Klopfleisch et al., 2010). We then characterized the antigenicity and immunogenicity of the recombinant adenovirus. Our study lays the foundation for a study of live-adenoviral–vectored FMDV vaccines.

## Materials and Methods

### Plasmids, Cells, Virus, and Animals

Plasmid pMD19-P12A3C (A/GDMM/CHA/2013), shuttle vector pAdTrack-CMV, human embryonic kidney 293 cells (HEK-293 cells), and the control adenovirus (WtAdv) are maintained in our laboratory. BALB/c mice and guinea pigs were provided by the Lanzhou Veterinary Research Institute (LVRI, Lanzhou, China) and handled in strict accordance with good animal practice according to the Animal Ethics Procedures and Guidelines of the People’s Republic of China, and the study was approved by the Animal Ethics Committee of LVRI, CAAS (no. LVRIAEC2017-003).

### Construction and Screening of Recombinant Adenoviruses

Using plasmid pMD19-P12A3C(A/GDMM/CHA/2013) as template, the region encoding P12A3C(meaning VP4-VP2-VP3-VP1-2A-3C FMDV sequences) of FMDV strain A/GDMM/CHA/2013 was amplified with polymerase chain reaction (PCR) using primers P12A-F (5′-GCCGAATTCATGGGGGCCGGGCAATCCAGCCCTGC-3′) and 3C-R (5′-GCCGCGGCCGCCTACTCGTGGTGTGGTTCAGGGTCGA-3′) and cloned into the multiple cloning site in the pAdTrack-CMV vector under the control of the cytomegalovirus promoter to generate pTrack-P12A3C^WT^-GD. There is a green fluorescent protein flag in the pAdTrack-CMV vector under the control of the cytomegalovirus promoter. Amino acid mutations G38S and F48S were introduced into the 3C region of pTrack-P12A3C^WT^-GD with the QuikChange II XL Site-Directed Mutagenesis Kit (Agilent, Santa Clara, CA, United States) to generate pTrack-P12A3C^G38SF48S^-GD. The recombinant vectors pTrack-P12A3C^WT^-GD and pTrack-P12A3C^G38SF48S^-GD were linearized by digestion with *Pme*I and used to transform competent *Escherichia coli* Ad-BJ5183 cells (maintained in our laboratory), which contain the plasmid pAdEasy-1, to produce pAd5-P12A3C^WT^-GD and pAd5-P12A3C^G38SF48S^-GD by homologous recombination. pAd5-P12A3C^WT^-GD and pAd5-P12A3C^G38SF48S^-GD were then linearized with *Pac*I and used to transfect HEK-293 cells with Lipofectamine 2000 (Thermo Fisher Scientific, Waltham, MA, United States) to generate the recombinant adenoviruses. The transfected HEK-293 cells were collected after 48 h and blindly passaged until an obvious cytopathic effect (CPE) and green fluorescence were observed. The recombinant adenoviruses were purified with plaque assays using HEK-293 cells. Then the purified adenoviruses were amplified by infecting HEK-293 cells at a multiplicity of infection of 5 pfu/cell. The supernatant cells were harvested after 48 h. The viruses were released by freeze/thaw cycles at −70°C and room temperature for three times. Viral titers were monitored with 50% tissue culture infective dose (TCID_50_) assays. The viral genomes were extracted and amplified with primers P12A-F and 3C-R to confirm that the target gene P12A3C was stably inherited in the recombinant adenoviruses. The WtAdv virus was used as the negative control.

### Analysis of Target Gene Expression in HEK-293 Cells

HEK-293 cells infected with rAdv-P12A3C^WT^-GD, rAdv-P12A3C^G38SF48S^-GD, or WtAdv were collected and used for Western blotting or an indirect sandwich enzyme-linked immunosorbent assay (IS-ELISA) to detect the expression of P12A3C, as previously described (Xie et al., 2016). A rabbit anti-FMDV polyclonal antibody directed against FMDV serotype A was used as a substitute for serotype O. Inactivated FMDV 146S of serotype A (Diagnostic Products Center, LVRI) was used as the positive control.

### Intramuscular and Intraocular–Nasal Immunization of Mice

Thirty-five 6-week-old female BALB/c mice were randomly divided into seven groups (*n* = 5 each) and immunized as described in Table 1. The mice were boosted with the same dose at 14 days postimmunization (dpi). Sera and nasal washes were collected at 0, 14, and 28 dpi. At 28 dpi, the mice immunized intramuscularly were killed, and their spleens were harvested for an enzyme-linked immunospot (ELISPOT) assay.

**TABLE 1:** Vaccination strategy in the mouse experiment.

Groups^a^	Treatments	Dose	Route	Priming week	Boosting week
a	rAdv-P12A3C^WT^-GD	1 × 10^8^ VP^b^	Intramuscular	0	2
b	rAdv-P12A3C^G38SF48S^-GD	1 × 10^8^ VP	Intramuscular	0	2
c	WtAdv	1 × 10^8^ VP	Intramuscular	0	2
d	inactivated vaccine^c^	100 μL	Intramuscular	0	2
e	rAdv-P12A3C^WT^-GD	1 × 10^8^ VP	Intraocular–nasal	0	2
f	rAdv-P12A3C^G38SF48S^-GD	1 × 10^8^ VP	Intraocular–nasal	0	2
g	WtAdv	1 × 10^8^ VP	Intraocular–nasal	0	2

*^*a*^Groups of immunized mice. ^*b*^VP, virus particles. ^*c*^Commercial inactivated type A FMDV vaccine containing ISA-206 adjuvant produced by China Agricultural Veterinarian Biology Science and Technology Co., Ltd., Lanzhou, China.*

### Detection of FMDV-Specific Antibodies and Cytokines

Foot-and-mouth disease virus–specific immunoglobulin G (IgG) antibodies in the sera from the intramuscularly immunized mice were detected with liquid-phase blocking ELISA (LPB-ELISA), and FMDV-specific IgA antibodies in the nasal washes of all the mice were detected with indirect ELISA, as previously described (Xie et al., 2016). A rabbit anti-FMDV polyclonal antibody against FMDV serotype A was used as a substitute for serotype O.

The splenocytes were isolated from the mouse spleens and the cytokines expressed by them were detected with a mouse IFN-γ precoated ELISPOT kit or mouse IL-4 precoated ELISPOT kit (Dakewe Biotech Company, Beijing, China), according to the manufacturer’s protocol, as previously described.

### Lymphocyte Proliferation Assay

A T-cell proliferation assay was performed on the splenocytes using a 3-(4,5-dimethylthiazol-2-yl)-2-5-diphenyltetrazolium bromide (MTT) assay. The cells were adjusted to a concentration of 2 × 10^6^ cells/mL and added to a 96-well plate at 100 μL per well. The cells were stimulated with concanavalin A (positive stimulus), inactivated FMDV 146S of serotype A, or RPMI 1640 medium (negative stimulus). The final concentration of each stimulus was 10 μg/mL. The stimulation index (SI) was the ratio of the optical density at a wavelength of 490 nm (OD_490_) of the stimulated group to that of the unstimulated group.

### Immunization and Challenge of Guinea Pigs

Twenty-eight 2-week-old guinea pigs were randomly divided into seven groups (*n* = 4 each) and immunized as described in Table 2. Sera and the nasal washes were collected at 0 and 25 dpi. The neutralizing antibodies in the sera of groups 1–4 were tested with a virus neutralization test (VNT; Xie et al., 2019). The FMDV-specific IgA antibodies in the nasal washes of all the guinea pigs were tested with indirect ELISA (Xie et al., 2016), as previously described. A rabbit anti-FMDV polyclonal antibody of serotype A was used as a substitute for serotype O. At 28 dpi, the guinea pigs were challenged with hundred 50% guinea pig infective doses of FMDV strain A/GDMM/CHA/2013 with an intradermal injection in the left rear foot.

**TABLE 2:** Vaccination strategy in the guinea pig experiment.

Groups^a^	Treatments	Dose	Route
1	rAdv-P12A3C^WT^-GD	1 × 10^9^ VP	Intramuscular
2	rAdv-P12A3C^G38SF48S^-GD	1 × 10^9^ VP	Intramuscular
3	WtAdv	1 × 10^9^ VP	Intramuscular
4	inactivated vaccine	200 μL	Intramuscular
5	rAdv-P12A3C^WT^-GD	1 × 10^9^ VP	Intraocular–nasal
6	rAdv-P12A3C^G38SF48S^-GD	1 × 10^9^ VP	Intraocular–nasal
7	WtAdv	1 × 10^9^ VP	Intraocular–nasal

*^*a*^Groups of immunized guinea pigs.*

### Statistical Analysis

Two groups were compared with an unpaired *t* test, and three groups were compared with one-way analysis of variance plus a Bonferroni posttest, in SPSS Statistics 2 (IMB, New York, United States). A significant difference was defined as *P* ≤ 0.05. ^∗^0.01 < *P* ≤ 0.05; ^∗∗^0.001 < *P* ≤ 0.01; ^∗∗∗^0.0001 < *P* ≤ 0.001.

## Results

### Construction and Characterization of Recombinant Adenoviruses

**FIGURE 1:**
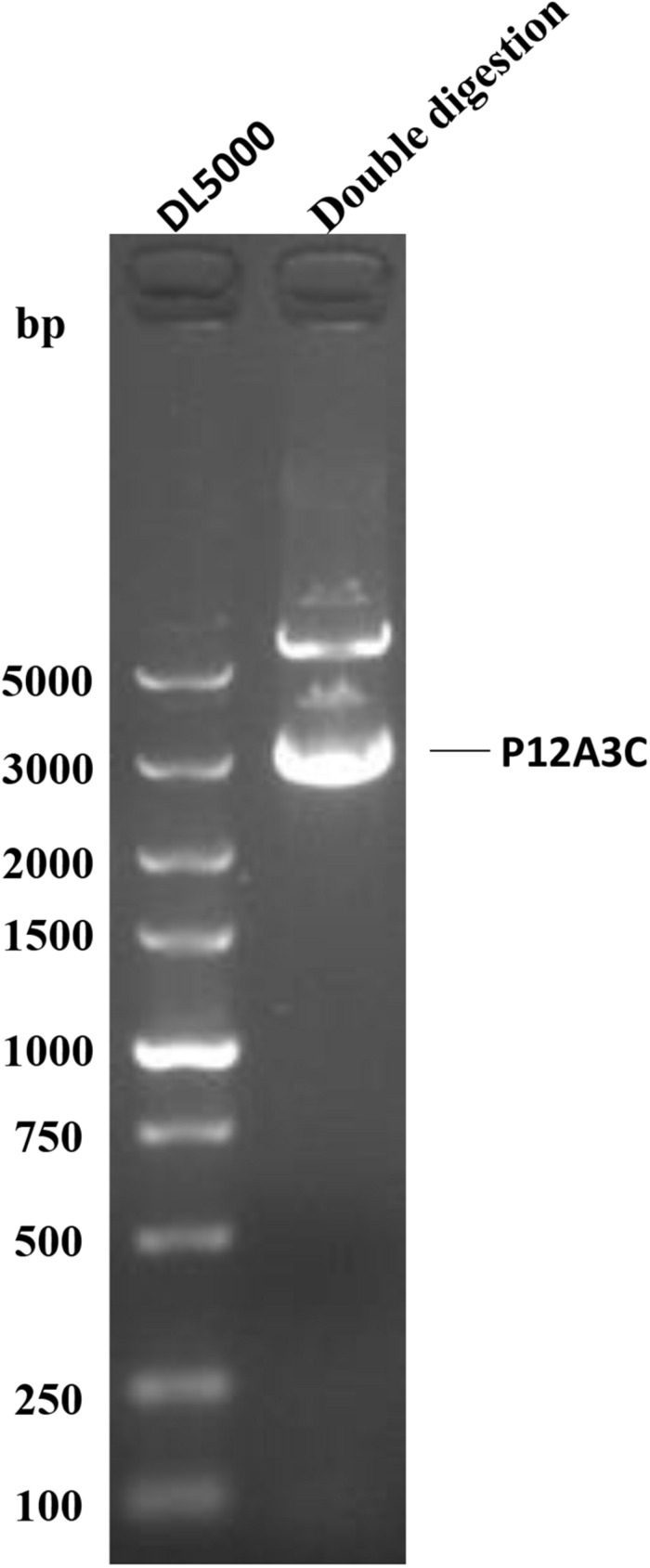
Identification of the shuttle vector with double enzymatic digestion.

**FIGURE 2:**
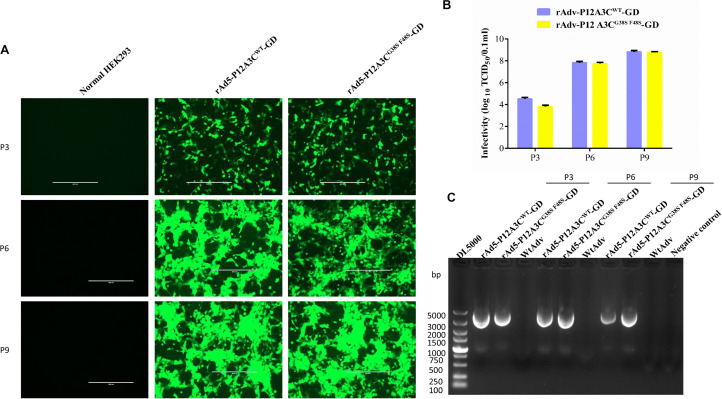
Construction and identification of the recombinant adenoviruses. **(A)** CPE and green fluorescence observed in different passages (P3, P6, and P9). **(B)** Adenoviral titers in different passages (P3, P6, and P9) were monitored with a TCID_50_ assay. Results are presented as means ± standard deviations (SD), *n* = 3. **(C)** Target gene P12A3C was amplified from different generations of recombinant adenoviruses.

To confirm the correct insertion of gene P12A3C, the positive clone pTrack-P12A3C^WT^-GD was identified with double enzymatic digestion. As shown in Figure 1, a fragment of approximately 3,000 bp, which was consistent with the target gene P12A3C, was generated. When blindly passaged to the third passage, an obvious CPE and green fluorescence were observed in pTrack-P12A3C^WT^-GD–transfected HEK-293 cells (Figure 2A), indicating that the recombinant adenoviruses were successfully constructed. The viral titers increased significantly when the recombinant adenoviruses were amplified on HEK-293 cells (Figure 2B). A PCR product of approximately 3,000 bp, which was consistent with the target gene P12A3C, was amplified from the recombinant adenovirus genome at different passages (P3, P6, and P9), whereas no fragment was amplified from WtAdv (Figure 2C), indicating that the target gene P12A3C was stably inherited by the recombinant adenoviruses.

### Expression and Antigenicity of the Recombinant Proteins

**FIGURE 3:**
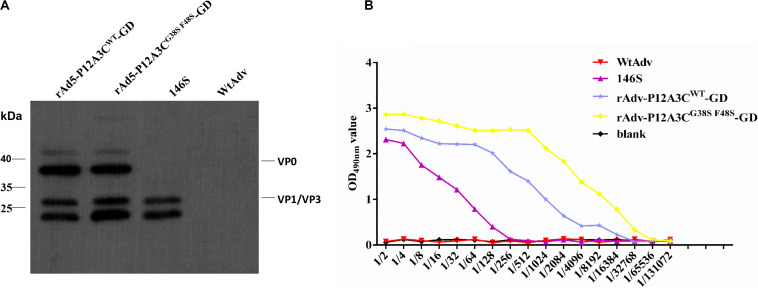
Identification of the recombinant proteins. **(A)** Western blotting analysis of the recombinant proteins. **(B)** Antigenicity of the recombinant proteins was tested with IS-ELISAs.

To confirm the correct expression of gene P12A3C, the target proteins were detected with Western blotting (Figure 3A) and IS-ELISAs (Figure 3B). Western blotting showed bands of approximately 25 kDa, corresponding to VP1/VP3 of rAdv-P12A3C^WT^-GD, rAdv-P12A3C^G38SF48S^-GD, and inactivated FMDV 146S and bands of 36 kDa corresponding to VP0 of rAdv-P12A3C^WT^-GD and rAdv-P12A3C^G38SF48S^-GD, but nothing was detected from WtAdv. The IS-ELISAs showed that the OD values for rAdv-P12A3C^WT^-GD and rAdv-P12A3C^G38SF48S^-GD decreased gradually with increasing dilution, consistent with the positive control 146S. However, the OD value of WtAdv was similar to that of the blank control. These results demonstrate that the recombinant adenoviruses efficiently expressed the target protein in HEK-293 cells.

### Intramuscular Immunization Induced High Anti-FMDV IgG Antibody Titers and Cellular Immune Responses in Mice

**FIGURE 4:**
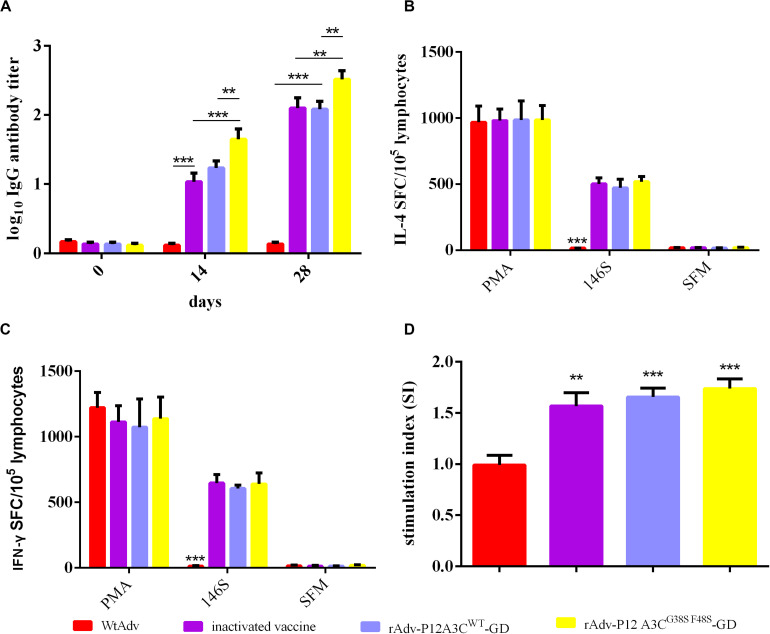
High anti-FMDV IgG antibody titers and cellular immune responses were induced in BALB/c mice. **(A)** Antibodies raised against serotype A FMDV were measured with LPB-ELISA. **(B)** Levels of mouse cytokine IL-4. The spot-forming cells (SFC) were counted after the plates were air-dried. **(C)** Levels of mouse cytokine IFN-γ. **(D)** Mean stimulation index (SI) of mice immunized with WtAdv, inactivated FMDV vaccine, rAdv-P12A3C^WT^-GD, or rAdv-P12A3C^G38SF48S^-GD at 28 dpi. Results are presented as means ± SD (*n* = 3).

Anti-FMDV IgG antibodies in the sera from the intramuscularly mice were detected with LPB-ELISA. In the groups immunized with the rAdv-P12A3C^WT^-GD, rAdv-P12A3C^G38SF48S^-GD, or FMD vaccine, all the mice produced high antibody titers against FMDV at 14 dpi compared with group WtAdv (*P* < 0.001), whereas rAdv-P12A3C^G38SF48S^-GD induced a higher antibody response than rAdv-P12A3C^WT^-GD or the inactivated FMDV vaccine (Figure 4A). These three groups induced the highest antibody responses at 28 dpi, and rAdv-P12A3C^G38SF48S^-GD induced a higher antibody titer than rAdv-P12A3C^WT^-GD or the inactivated FMDV vaccine (*P* < 0.01). These results suggested that the rAdv-P12A3C^WT^-GD and rAdv-P12A3C^G38SF48S^-GD vaccines induce antibodies against FMDV.

Cytokines IFN-γ and IL-4 were detected with ELISPOT assays, after the mice were immunized with inactivated FMDV 146S, rAdv-P12A3C^WT^-GD, rAdv-P12A3C^G38SF48S^-GD, or the inactivated FMDV vaccine, which all induced higher concentrations of IFN-γ and IL-4 than were detected in the group vaccinated with WtAdv (*P* < 0.001; Figures 4B,C). The T-cell proliferative responses were evaluated with an MTT assay. The mouse groups treated with rAdv-P12A3C^WT^-GD, rAdv-P12A3C^G38SF48S^-GD, or the inactivated FMDV vaccine showed significantly higher T-lymphocyte proliferation than the WtAdv-treated group (Figure 4D). These results suggested that the recombinant adenoviruses induced an effective cellular immune response against FMDV.

### The Recombinant Adenoviruses Induced High Anti-FMDV IgA Antibody Titers in Mice

**FIGURE 5:**
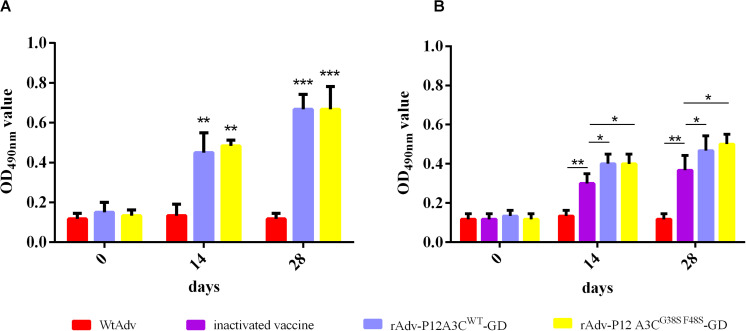
Anti-FMDV IgA antibodies detected with indirect ELISA. **(A)** Anti-FMDV IgA antibodies in the nasal washes of the intraocular–nasally immunized mice were tested with indirect ELISA. **(B)** Anti-FMDV IgA antibodies in the nasal washes of the intramuscularly immunized mice were tested with indirect ELISA. Results are presented as means ± SD (*n* = 3).

The nasal washes of the immunized mice were tested for anti-FMDV IgA antibodies with an indirect ELISA after they were diluted 1:50 or 1:4. The net anti-FMDV responses in the samples are shown as mean ODs. As shown in Figure 5, both rAdv-P12A3C^WT^-GD and rAdv-P12A3C^G38SF48S^-GD induced high IgA antibody responses than WtAdv (*P* < 0.01) at 14 dpi, and even higher IgA antibody responses were induced at 28 dpi. These result suggested that rAdv-P12A3C^WT^-GD and rAdv-P12A3C^G38SF48S^-GD induced IgA antibodies against FMDV.

### Recombinant Adenoviruses Protect Guinea Pigs Against FMDV

**FIGURE 6:**
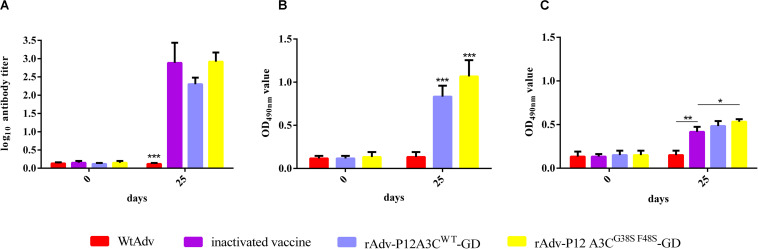
High neutralizing antibody titers and FMDV-specific IgA antibodies were induced in guinea pigs. **(A)** Neutralizing antibodies in the sera of the intramuscularly immunized guinea pigs were tested with VNT. **(B)** Anti-FMDV IgA antibodies in the nasal washes of the intraocular–nasally immunized guinea pigs were tested with indirect ELISA. **(C)** Anti-FMDV IgA antibodies in the nasal washes of the intramuscularly immunized guinea pigs were tested with indirect ELISA. Results are presented as means ± SD (*n* = 3).

The neutralizing antibodies in the sera of the intramuscular immunization guinea pigs were tested with VNT. The neutralizing titers were very low in all four groups of guinea pigs at 0 dpi, and there was no significant difference between the four groups (Figure 6A). At 25 dpi, higher levels of neutralizing antibodies were induced by the inactivated vaccine, rAdv-P12A3C^WT^-GD, and rAdv-P12A3C^G38SF48S^-GD than WtAdv did (*P* < 0.001). The nasal washes of the immunized guinea pigs were tested for anti-FMDV IgA antibodies with an indirect ELISA (Figures 6B,C). Both rAdv-P12A3C^WT^-GD and rAdv-P12A3C^G38SF48S^-GD induced higher IgA antibody responses at 25 dpi than WtAdv did (*P* < 0.01).

After challenge with strain A/GDMM/CHA/2013 at 28 dpi, the guinea pigs were monitored for lesions on the footpads for 10 consecutive days postchallenge (dpc) and were then euthanized on day 11 (or before in cases of severe signs of disease). All the guinea pigs in groups 3 and 7 developed lesions on the uninjected footpads at 2 dpc, as shown in Table 3. At 3 dpc, two guinea pigs developed lesions on the uninjected footpads in group 5. At 4 dpc, one guinea pig in group 1 and two guinea pigs in group 6 developed lesions on the uninjected footpads. At 5 dpc, one guinea pig developed lesions on the uninjected footpads in group 5. None of the guinea pigs in the other two groups developed severe vesicles. Unprotected guinea pigs showed signs of emaciation, lethargy, rough fur, and blisters. These results indicate that rAdv-P12A3C^G38SF48S^-GD protected 100% of guinea pigs against FMDV when administered intramuscularly.

**TABLE 3:** Protection of and symptom severity in guinea pigs after challenge with A/GDMM/CHA/2013 strain.

Serial number^b^	Groups^a^						
	1	2	3	4	5	6	7
**Protection**							
1	Pro^c^	Pro	N-Pro	Pro	N-Pro	N-Pro	N-Pro
2	N-Pro^d^	Pro	N-Pro	Pro	N-Pro	Pro	N-Pro
3	Pro	Pro	N-Pro	Pro	Pro	N-Pro	N-Pro
4	Pro	Pro	N-Pro	Pro	N-Pro	Pro	N-Pro
**Severity of symptoms**							
1	None	None	Severe	None	Severe	Severe	Severe
2	Severe	None	Severe	None	Severe	None	Severe
3	None	None	Severe	None	None	Severe	Severe
4	None	None	Severe	None	Severe	None	Severe
Rate of protection (%)	75 (3/4)	100 (4/4)	0(0/4)	100 (4/4)	25 (1/4)	50 (2/4)	0 (0/4)

*^*a*^Groups of the immunized guinea pigs. ^*b*^Serial number of immunized guinea pigs in each group. ^*c*^Protection, indicating no lesions on footpads other than the inoculated footpad. ^*d*^Nonprotection, indicating the appearance of a secondary lesion on any footpad other than the inoculated footpad.*

## Discussion

An inactivated FMDV vaccine has played a significant role in the prevention and control of FMDV (Caridi et al., 2017; Khalifa et al., 2017). However, the traditional inactivated vaccine has many disadvantages. Therefore, the development of alternative vaccines has been extensively explored. Several studies have examined the protective effects of recombinant adenoviruses expressing the FMDV P12A and 3C proteins of different serotypes (Zhou et al., 2013; Kumar et al., 2015; Xie et al., 2016; Sreenivasa et al., 2017). However, the endemic strain in China, FMDV A/GDMM/CHA/2013, has rarely been investigated. Given the extremely weak cross-immunity conferred by the FMDV subtypes, we expressed the P12A and 3C proteins of FMDV strain A/GDMM/CHA/2013 in an adenoviral vector in this study.

When blindly passaged to the third passage, an obvious CPE and green fluorescence (Figure 2A) were observed in HEK-293 cells. The viral titers increased significantly with the passage of the viruses (Figure 2B), and we successfully amplified the target gene P12A3C from different passages (P3, P6, and P9). These results suggested that the recombinant adenoviruses rAdv-P12A3C^WT^-GD and rAdv-P12A3C^G38SF48S^-GD were successfully constructed and that the target gene is inherited stably by the recombinant adenoviruses. Mutations G38S and F48S were induced in 3C to reduce its protease activity. The same bands for VP0 and VP1/VP3 were detected in rAdv-P12A3C^WT^-GD and rAdv-P12A3C^G38SF48S^-GD, suggesting that both the wild-type 3C and mutant 3C proteins cleave P1-2A to generate VP0, VP3, and VP1. However, rAdv-P12A3C^G38SF48S^-GD showed a higher OD_490_ value and better immunogenicity than rAdv-P12A3C^WT^-GD, suggesting that the mutation of 3C reduced the cellular toxicity of the construct and increased the yield of structural proteins.

Both the humoral and cellular immune responses play important roles in the fight against FMDV infection. Therefore, in this study, anti-FMDV IgG antibodies and cytokines were detected to evaluate the immunogenic effects of the recombinant adenoviruses. All the mice secreted anti-FMDV IgG antibodies directed against FMDV at 14 and 28 dpi and expressed high levels of IL-4 and IFN-γ at 28 dpi. These results suggest that recombinant adenoviruses rAdv-P12A3C^WT^-GD and rAdv-P12A3C^G38SF48S^-GD stimulate strong T-cell responses and humoral immune responses to FMDV.

Foot-and-mouth disease virus infects its hosts through the respiratory mucosa. The production of secretory IgA in the respiratory mucosa blocks the FMDV infection process at its inception. Therefore, in this study, the BALB/c mice and guinea pigs were immunized with the recombinant adenoviruses via an intraocular–nasal route to test whether secretory IgA is induced by recombinant adenoviruses rAdv-P12A3C^WT^-GD and rAdv-P12A3C^G38SF48S^-GD. The nasal washes were tested for anti-FMDV IgA antibodies with an indirect ELISA. The results showed that high levels of secretory IgA antibodies specific for FMDV were induced in the immunized BALB/c mice and guinea pigs, demonstrating that recombinant adenoviruses rAdv-P12A3C^WT^-GD and rAdv-P12A3C^G38SF48S^-GD block FMDV invasion of the respiratory mucosa.

Neutralizing antibodies play a critical role in protecting animals from FMDV, so we detected the neutralizing antibody titers induced in guinea pigs by intramuscular immunization. At 0 dpi, the neutralizing titers were very low in all four groups, at 0.12–0.15 log_10_. At 25 dpi, high levels of neutralizing antibodies (2.3–2.9 log_10_) were induced by the inactivated vaccine, rAdv-P12A3C^WT^-GD, and rAdv-P12A3C^G38SF48S^-GD, whereas the antibody titer in group WtAdv was 0.12 log_10_. rAdv-P12A3C^G38SF48S^-GD protected all the guinea pigs against challenge with strain A/GDMM/CHA/2013, whereas rAdv-P12A3C^WT^-GD protected 75% of the guinea pigs against challenge.

In summary, we successfully expressed the P12A and mutated 3C proteins of FMDV strain A/GDMM/CHA/2013 in a replication-deficient human adenovirus type 5 vector. The target genes were inherited stably by the recombinant adenoviruses. Recombinant adenovirus rAdv-P12A3C^G38SF48S^-GD showed better immunogenicity than rAdv-P12A3C^WT^-GD. All the guinea pigs were protected by rAdv-P12A3C^G38SF48S^-GD against challenge with strain A/GDMM/CHA/2013 when immunized intramuscularly. Our results demonstrated the potential utility of the recombinant adenovirus rAdv-P12A3C^G38SF48S^-GD as an alternative vaccine to block the spread of FMDV strain A/GDMM/CHA/2013 in our country.

## Data Availability Statement

The raw data supporting the conclusions of this article will be made available by the authors, without undue reservation, to any qualified researcher.

## Ethics Statement

BALB/c mice and guinea pigs were provided by the LVRI and handled in strict accordance with good animal practice according to the Animal Ethics Procedures and Guidelines of the People’s Republic of China, and the study was approved by the Animal Ethics Committee of LVRI, CAAS (No. LVRIAEC2017-003).

## Author Contributions

YX performed experiments. YX, ZL, and HC analyzed the data. All authors conceived and designed the experiments, wrote, read, and critically reviewed the manuscript.

## Conflict of Interest

The authors declare that the research was conducted in the absence of any commercial or financial relationships that could be construed as a potential conflict of interest.
